# Season and Weather Effects on Travel-Related Mood and Travel Satisfaction

**DOI:** 10.3389/fpsyg.2017.00140

**Published:** 2017-02-06

**Authors:** Dick Ettema, Margareta Friman, Lars E. Olsson, Tommy Gärling

**Affiliations:** ^1^Faculty of Geosciences, Utrecht UniversityUtrecht, Netherlands; ^2^Service Research Center (CTF) and Samot VINN Excellence Center, Karlstad UniversityKarlstad, Sweden; ^3^Department of Economics, University of GothenburgGothenburg, Sweden

**Keywords:** everyday travel, season, weather, travel-related mood, travel satisfaction, travel mode

## Abstract

This study examines the effects of season and weather on mood (valence and activation) and travel satisfaction (measured by the Satisfaction with Travel Scale). Analyses are presented of 562 time-sampled morning commutes to work made by 363 randomly sampled people in three different Swedish cities asking them to use smartphones to report their mood in their home before and directly after the commutes. These reports as well as satisfaction with the commute obtained in summer and winter are linked to weather data and analyzed by means of fixed-effects regression analyses. The results reveal main effects of weather (temperature and precipitation) on mood and travel satisfaction (temperature, sunshine, precipitation, and wind speed). The effects of weather on mood and travel satisfaction differ depending on travel mode. Temperature leads to a more positive mood, wind leads to higher activation for public transport users, and sunshine leads to a more negative mood for cyclists and pedestrians. Sunshine and higher temperatures make travel more relaxed although not for cycling and walking, and rain and snow lead to a higher cognitive assessed quality of travel.

## Introduction

Influences of atmospheric factors on human behavior was a decade or more ago a topical research interest in environmental psychology (e.g., Suedfelt, 1987), in particular spurred by early field observations that rapes and murders increase in hot temperatures and decrease in cold temperatures (Anderson et al., 2000; Rotton and Cohn, 2003). Although this interest seems to have declined in current research (Gifford, 2014), many questions are still in need of being addressed. By atmospheric factors we primarily refer here to outdoor temperatures, sunshine, wind speed, and precipitation of which people are aware. Because of this awareness, they are likely to make choices that mediate influences of the atmospheric factors. An example is choice of spending leisure time outdoors when it is warm and sunny. Another atmospheric factor likely to also influence people's choices is hours of daylight that in many countries vary with season.

Positive and negative moods are also known to be influenced by atmospheric factors. Kämpfer and Mutz (2013) found an influence of sunshine on life satisfaction and argued that this effect is mediated by mood (defined as frequencies of experienced positive vs. negative emotions). They reasoned that sunshine activates positive emotions due to an increase of serotonin in the brain. Kööts et al. (2011) used an experience sampling method to measure mood on-line, finding that warmer temperatures increase the frequency of both positive and negative emotions, whereas higher humidity has the reverse effect. Studies conducted in hot climates show that high temperatures are disadvantageous for physical activities (e.g., Tu et al., 2004; Miranda-Moreno and Nosal, 2011). Elderly seems to be more affected by temperature and being outdoors increases the influence of weather on mood (Kööts et al., 2011). By comparing mood experienced at average daily temperatures (10–16°C or 50–60°F) with temperatures above 70°F (21°C), Noelke et al. (2016) found that high temperatures reduce positive emotions (e.g., joy, happiness), increase negative emotions (e.g., stress, anger), and increase fatigue (e.g., feeling tired, low energy). These effects were found to be particularly strong among less educated and older people with more limited possibilities to protect themselves from high temperatures. Denissen et al. (2008) reported that warmer temperatures and sunshine increase both positive and negative mood. They also found that a higher wind speed leads to both lower positive and negative mood. Weather still appears to explain only a small percent of the variance in mood, possibly due to substantial individual differences in how mood is influenced by weather (Klimstra et al., 2011). In line with this, Connolly (2013) report that women are more responsive than men to different weather conditions.

Lucas and Lawless (2013) identified ten studies that examined the association between season and mood. They conclude that some associations have been found, but that the weather conditions that appear to be most important vary across studies, and that some studies have found no effects. For instance, Keller et al. (2005) found that during spring time, higher temperatures, and higher barometric pressures correlate with positive mood, particularly if time is spent outdoors. During the summer, however, higher temperatures exceeding comfort thresholds lead to more negative moods. Seasonal changes in hours of sunshine are found to account for relationships between weather and variability in mood (Beecher et al., 2016) with negative feelings of distress reported to increase during seasons with shorter hours.

While the cited studies have investigated the relationship between weather and season on mood in general (i.e., averaged across a variety of contexts), how seasonal differences and weather influence satisfaction with various everyday activities have not been investigated. Travel is an important instrumental activity that most people undertake most of the days. The commute to and from work is the most frequent type of travel, worldwide undertaken by billions of people every weekday. Satisfaction with, as well as mood influences of the commute trip, may differ in different seasons depending on the weather. Furthermore, different travel modes may have different influences on the exposure to weather conditions. Pedestrians and cyclists are directly exposed during the whole journey, whereas public transport users are exposed during access and egress. Drivers and passengers of automobiles are influenced by weather-related driving conditions (e.g., poor sight and slippery surface).

In this paper we report both seasonal and weather effects on on-line measures of mood before and after work commutes by different travel modes in three different cities in Sweden, as well as retrospective satisfaction with the work commutes. Sweden is characterized by cold temperatures in winter (on average between −15 and 0°C) and mild or warm temperatures in summer (on average between 10 and 19°C). Hours of daylight also differ substantially in winter (from an average of 0–9 h) and summer (from an average of 19–24 h). Justifying that both seasonal and weather effects are measured, temperatures, sunshine, wind speed, and precipitation vary substantially within seasons. Furthermore, only rainfalls are prevalent in summer, whereas both rainfalls and snowstorms are prevalent in winter.

The paper proceeds by first reviewing previous research investigating satisfaction with work commutes, followed by a review of previous research of mood effects of work commutes. A third section then reviews the scant research of seasonal and weather effects on travel-related moods and satisfaction with travel. Our empirical study is reported last.

### Review of previous research

#### Travel satisfaction

Travel satisfaction is defined as a domain-specific satisfaction measure based on how travelers evaluate daily travel conditions. Abou-Zeid et al. (2012) used ratings of satisfaction on a single scale to directly measure to what extent travelers are satisfied with their travel, usually referring to a particular journey or trip. Jakobsson Bergstad et al. (2011) used five scales which primarily tapped a cognitive evaluation. A more elaborated set of scales labeled Satisfaction with Travel Scale (STS) was later developed by Ettema et al. (2011) and Friman et al. (2013). The STS is analogous to existing scales for measuring subjective well-being (SWB).

Conceptualizations of SWB (Diener and Suh, 1997; see review in Busseri and Sadava, 2011) posit three different components: a cognitive evaluation of life satisfaction and two affective evaluations consisting of the frequency of positive emotions (or positive affect: PA) and the frequency of negative emotions (or negative affect: NA) experienced during a past time interval. Analogously, Friman et al.'s (2013) STS include three scales to tap a cognitive evaluation of travel and six scales to tap affective evaluations. The latter scales were chosen to represent combinations of the two core affect dimensions of valence and activation (Russell, 1980, 2003; Västfjäll et al., 2002). Three scales cover experience of travel on a dimension from negative deactivation (e.g., boredom) to positive activation (e.g., enthusiasm), and three scales experience of travel on a dimension from negative activation (e.g., stress) to positive deactivation (e.g., feeling relaxed). The STS has been applied in various geographical settings (e.g., Sweden, The Netherlands, Belgium, and Japan) and has led to consistent and interpretable outcomes.

Empirical studies of travel satisfaction throughout the world (e.g., Gatersleben and Uzzell, 2007; Olsson et al., 2013; Martin et al., 2014; St-Louis et al., 2014) consistently show that satisfaction with travel by slow modes (walking, cycling) is higher than travel by car. Public transport consistently leads to lower satisfaction than car. However, Martin et al. (2014) observed in their longitudinal 10-year study, a positive association between well-being and public transport when compared to car travel. Furthermore, this observed association was of comparable magnitude to that observed between well-being and travel by slow modes (also referred to as active travel). Satisfaction with car trips has been found to be negatively affected by long distances and congestion (e.g., Novaco et al., 1990; Stutzer and Frey, 2008; see review in Novaco and Gonzales, 2009). Experienced traffic risks, annoyance with other road users, the trip being tiring, being distracted by billboards, and lack of freedom to choose speed and lane are additional factors influencing car drivers negatively (Ettema et al., 2013). Satisfaction with public transport has been found to be influenced by the opportunity to carry out activities on board (Ettema et al., 2012), by unexpected negative critical incidents such as delays (Friman and Gärling, 2001) as well as service-related factors such as crowdedness, reliability, frequency, fares, vehicle comfort, convenience, and access (Redman et al., 2013). Satisfaction with walking correlates negatively with duration (St-Louis et al., 2014) and positively with liveliness of the environment and the presence of other people (Ettema and Smajic, 2015). Extensive research investigating the factors promoting walking and cycling (but not directly travelers' satisfaction; see Ettema et al., 2016, for review) suggest the importance of factors such as quality of infrastructure (e.g., separate cycling lanes), crossings, slope, parking facilities (for cyclists), and esthetics of the environment influence walking and cycling frequency. However, the direct impact of these factors on travel satisfaction has not been fully investigated.

### Mood effects of travel

Travel satisfaction cannot be directly equated with the emotional well-being effect of travel, as assessed by travelers' mood after travel. Mood is defined as a prolonged core affect (Russell, 2003) that would be assessed by the affective SWB dimensions. Mood is lingering for a longer time, but can change due to emotional responses to the (travel) environment. According to Gärling et al., Unpublished manuscript, an event (such as a trip or an incident during a trip) will have a larger impact on mood if the emotional impact of the event is stronger due to personal relevance. For example, the impact of a delay on mood will be larger if travelers anticipate to be late for an important meeting than when making a leisure trip, since emotional distress due the consequences of the delay will be larger. Thus, the impact of travel conditions on travel satisfaction and on mood may differ, depending of the salience of the travel conditions to the traveler.

A limited number of studies have investigated mood effects of travel. In Kahneman et al. (2004) commuting was found to be associated with negative mood in retrospective measures of emotional responses to daily episodes. Morris and Guerra (2014) analyzed data from a large US sample. A retrospective aggregated measure was obtained of mood (based on 0–6 ratings of happiness, sadness, tiredness, pain, and stress) experienced during the preceding day. Excluding purely recreational travel, the results showed that daily travel only accounted for a few percent of the mood variance. This was still not a trivial effect compared to several other activities included in the same study. Jakobsson Bergstad et al. (2011) showed a direct effect of satisfaction with travel on a retrospective measure of the weekly affect balance as well as an indirect effect through affect associated with performance of frequent travel-related out-of-home activities during the week. Olsson et al. (2013) found that retrospectively measured positive affect decreased with the duration of work commutes. Feng and Boyle (2014) analyzed data from a large-scale British study concluding that long work commutes are associated with negative mood more strongly for women than men. A higher load of household duties and more trip chaining were proposed explanations of the observed sex difference.

Friman et al., (submitted) used a smartphone application to record mood on-line. They found that mood immediately following a trip was influenced by positive and negative incidents during the trip and marginally by delays during the trip. However, mood later after the trip was not influenced by these factors, but was affected by travel time, which thus had a longer-term impact. The same study showed that travel satisfaction (i.e., the assessment of the trip itself) was influenced by a wider set of trip attributes than mood, providing support for that mood and travel satisfaction are only partially related.

### Weather and season effects on travel

Weather and season effects on travel are increasingly being investigated (see Böcker et al., 2013). For instance, Böcker and Thorsson (2014) found that cycling frequency is negatively affected by precipitation and wind speed, and that the highest frequency is observed for an air temperature of 24°C. Yet, the effects of weather and season on travel-related mood and travel satisfaction have received only limited attention. Böcker et al. (2013) found lower mood levels for pedestrians and cyclists under dark and shimmery conditions, with temperatures above 25°C, and rainy conditions. St-Louis et al. (2014) found that season effects play a role for satisfaction with slow modes. Both walking and cycling yield lower satisfaction in the Canadian cold snowy season, with cyclists being affected most. Stradling et al. (2007) found that the availability of protection against weather influences satisfaction with bus services, but they did not investigate influences of the weather *per se*.

### Research questions

Although existing research has investigated seasonal or weather effects on mood and to a lesser extent on travel satisfaction, no studies have been made of both seasonal and weather effects on travel-related mood. As indicated by our theoretical propositions (Gärling et al., Unpublished manuscript) and empirical results (Friman et al., Submitted), the effects on mood after travel may differ from the effects on travel satisfaction. In addition, jointly investigating season and weather is important since the weather may vary widely within a season. Our empirical study therefore addresses the question of to what extent season and weather influence both travel-related mood and travel satisfaction. We expect a more negative mood in winter (with less hours of sunshine) than in summer, and, within seasons, that mood is more negative when it is cold, windy, and raining or snowing. Mood effects of travel as well as satisfaction with travel are likewise expected to be more negative in winter than in summer, and, within seasons, more negative when it is cold, windy, and raining or snowing. The negative effects on mood and travel satisfaction in winter as compared to summer as well as within seasons should in particular be observed for people who walk or bike which otherwise would lead to a more positive mood and higher satisfaction. Also people using public transport may be more affected since they are less protected from the weather than those who use cars. Drivers and passengers of cars may be influenced by weather-related driving conditions.

## Materials and methods

### Sample

A recruitment company enrolled randomly sampled participants from three urban areas in Sweden (Stockholm, pop. appr. 1.400,000; Göteborg, pop. appr. 750,000; Karlstad, pop. appr. 100,000) located at approximately the same latitude (59.3–57.7) and longitude (18.1–12.0). The sample was stratified such that equal numbers were targeted from each urban area, for the primary travel modes of car, public transport, and cycling or walking (defined as slow modes), and sex. This procedure was followed during the winter (February 2–27) as well as during the summer (June 4–18) resulting in data from two independent subsamples. Sample descriptive are given in Table 1.

**Table 1:** **Sample descriptive**.

	**Winter (*n* = 188)**	**Summer (*n* = 175)**
**LOCATION**		
Stockholm	48 (25.5%)	53 (30.3%)
Göteborg	73 (38.8%)	65 (37.1%)
Karlstad	67 (36.5%)	57 (32.6%)
**SEX**		
Male	64 (34.0%)	78 (44.6%)
Female	124 (66.0%)	97 (55.4%)
Age (mean and s.d.)	40.0 (13.1)	41.9 (10.9)
**HOUSEHOLD TYPE (FREQUENCY)**		
Single	46 (25.6%)	25 (17.5%)
Single parent	12 (6,7%)	15 (10.5%)
Couple without children	40 (22.2%)	26 (18.2%)
Couple with children	82 (45.6%)	77 (53.8%)
**INCOME (SEK/MONTH)**		
<20,000	37 (20.6%)	10 (7.0%)
20–25,000	23 (12.8%)	13 (9.1%)
25–30,000	32 (17.8%)	35 (24.5%)
30–35,000	38 (21.1%)	23 (16.1%)
35–40,000	24 (13.3%)	19 (13.3%)
>40,000	26 (14.4%)	43 (30.1%)

The table indicates that participants are equally distributed across season, subsamples and locations. Women are slightly overrepresented (60.9%), in particular in the winter subsample. Households with children account for about half of the sample. Single parents account for the smallest share of household types. In terms of income distribution, all income levels are represented. However, lower income participants are overrepresented in the winter subsample and higher income participants are overrepresented in summer subsample.

Participants were recruited 1 or 2 weeks before the planned data collection. The weather conditions differed between the two waves. In winter morning temperatures varied between −1.1 and 8.2°C (Stockholm between 1.5 and 7.3°C, Göteborg between 2.4 and 8.2°C, and Karlstad between −1.1 and 6.3°C). Daylight started around 7.30 am and ended around 5.00 pm. It was raining (and occasionally snowing) during 32% of the days. In summer morning temperatures varied between 10.4 and 19.7°C (Stockholm between 11.4 and 19.7°C, Göteborg between 12.3 and 16.2°C, and Karlstad between 10.4 and 17.6°C). Daylight started around 3.30 am and ended around 10.00 pm. It was raining during 26% of the days.

### Procedure^1^

In the recruitment interviews participants were given instructions of how to download from AppStore or Google Play the application MyExperience that was later used to send questionnaires to their smartphones at different times during a day. A total of 199 participants agreed to answer questions during 2 days, and another 164 participants to answer questions during one day.

Participants reported in the recruitment interviews which days they would commute to work during the following 2 weeks, the travel modes they would use, approximate departure times, and commute durations. This information was used when distributing the questionnaires through MyExperience. Questionnaires were sent at time *T*_*0*_ 30–60 min before the expected time of the morning commute, and at time *T*_*1*_ directly after the commute^2^. The participants were notified when a new questionnaire was available, along with information about when to answer it. If participants did not answer the questionnaire, a reminder was sent 30 min after *T*_*0*_ and *T*_*1*_, respectively. Each question in the smartphone questionnaire was answered on a separate page. For those participating 2 days the same procedure was repeated the second day. After the last smartphone questionnaire had been answered, a survey was sent by e-mail including questions about income, household composition, age, gender, travel habits, experience with travel in general, emotional well-being during the last month, and overall life satisfaction. Only answers to the questions about socio-demographics are reported here.

### Measurement instruments

#### Mood

Mood was measured with the question “How do you feel right now?” answered by means of ratings on two bipolar 7-point scales adapted from the Swedish Core Affect Scale or SCAS (Västfjäll et al., 2002; Västfjäll and Gärling, 2007), one for valence with the left end-point “Very sad, depressed, displeased” (−3) and the right end-point “Very glad, joyful, pleased” (3), the other for activation with the left end-point “Very passive, sleepy, dull” (−3) and the right end-point “Very active, awake, peppy” (3) with 0 as a neutral point. The ratings of mood were made at time *T*_*0*_ and *T*_*1*_, and always before answering any other questions.

#### Satisfaction with travel

A shortened STS (Friman et al., 2013) with three scales was used to measure travel satisfaction.^3^ Each scale was defined by the three pairs of end-point statements that in STS define three separate scales. The question “How did you experience your trip?” was posed and answered by means of ratings on bipolar 7-point scales from −3 to 3 tapping the cognitive evaluation (CE) with the left end-point jointly defined by “The trip worked very poorly, held low standard, was the worst imaginable” and the right end-point jointly defined by “The trip worked very well, held high standard, was the best imaginable,” the feeling of enthusiasm vs. boredom (PAND) with the left end-point jointly defined by “I felt very bored, tired, fed-up” and the right end-point jointly defined by “I felt very enthusiastic, alert, engaged,” and the feeling of relaxation vs. stress (PDNA) with the left end-point jointly defined by “I felt very stressed, worried, hurried” and the right end-point jointly defined by “I felt very relaxed, calm, confident.” STS was administered directly after the mood ratings and only at time *T*_*1*_.

#### Commute attributes

At time *T*_*1*_ questions were last asked about the commute. Participants indicated what primary mode they had used (car as driver, car as passenger, motorcycle, train, tram, subway, bus, cycle, or walking) with the possibility to add an additional mode if none was applicable. Although only season and weather effects on travel mode will be analyzed, participants also reported duration in minutes of the total commute, any delays in minutes, whether they were alone or accompanied by one or several family members, friends, or co-workers, and whether they had experienced any critical (unexpected) incident evoking negative or positive emotional responses. If an incident had occurred, they specified its nature in an open-ended answer box for positive emotional responses and another for negative emotional responses.

#### Weather variables

Data for precipitation, temperature, sunshine, and wind speed were obtained from RL.SE (https://rl.se/vadret) that merge data from two large data sources: LFV—Air Navigation Services of Sweden (Sweden), and NOAA—National Oceanic and Atmospheric Administration (U.S.). With respect to resolution of the data, all variables were collected on a half hour basis from weather stations at the airports of the cities of Karlstad, Göteborg (Säve) and Stockholm (Bromma). Precipitation is represented by a dummy (Rain/snow > 1 mm = 1; No rain/snow = 0), sunshine by another dummy (clear sky = 1; occasional clouds, scattered skies, broken clouds, or cloudy conditions = 0), still another dummy for wind speed (if > 4 m/s = 1; otherwise = 0), and temperature in degree Celsius subtracted by the mean for the month. The threshold values were based on the distribution of amount of rain/snow and wind speed across observations in our sample. 71.2% of observations had no rain and 6.2% <1 mm of rain per half hour, which can be considered drizzle. Observations with more than 1 mm of rain per half hour faced continuous rain, which we interpret as rainy conditions. More refined classifications were not possible given the low number of observations with rain. Regarding wind force, a cut off level of 4 m/s was chosen given that it separates the 64.2% least windy conditions, up to a gentle breeze. A detailed description of the distributions of the weather variables parameters across season is found in the Appendix (Tables A1–A3) in Supplementary material.

## Results

### Travel-related mood

In order to investigate the effects of season and weather factors on mood directly after a commute, we estimated fixed effects regression analyses^4^ of mood at *T*_*1*_. Separate models are estimated for valence and activation. Since some participants answered the questionnaire on multiple days, the analyses distinguish between variance across observations and across individuals. In this way, we control for unobserved individual differences that may affect mood ratings. Explanatory variables in the models are individual characteristics, season (winter vs. summer) and weather conditions (relative daily temperature, whether it rained/snowed or not, whether it was windy or not, and whether it was sunny or cloudy). Since mood has many other known determinants than daily travel, we included mood prior to the commute (measured at *T*_*0*_) as another explanatory variable. As a consequence, we estimate the effects of the commute on mood changes. In the models, we also test whether interactions between weather conditions, season and travel mode significantly influence mood. We test two-way interactions (season × travel mode, weather × travel mode,) and three-way interactions (season × weather × travel mode). In the final model, we retained all main effects of weather and season but only two-way and three-way interactions that were significant at *p* < 0.10.

Table 2 shows that mood prior to the commute (valence and activation at *T*_*0*_) is influenced by age and sex. Consistent with previous research, being older is associated with a positive mood. The marginally significant effect of sex suggests that in the morning women are slightly in a more positive mood than men are. Sunshine is associated with a higher valence in the morning, although this effect is only marginally significant.

**Table 2:** **Unstandardized coefficients from the fixed-effects regression analyses of mood before (*T*_*0*_) and directly after (*T*_*1*_) the commute**.

	**Mood before the commute**		**Mood directly after the commute**	
	**Valence *T_0_* M *(SD)***	**Activation *T_*0*_* M *(SD)***	**Valence *T_*1*_* M *(SD)***	**Activation *T_*1*_* M *(SD)***
Predictor	0.21 (1.45)	1.18 (1.09)	1.10 (1.19)	0.91 (1.30)
Intercept	0.529^**^	−0.924^**^	1.126^***^	1.160^***^
Valence T_0_			0.431^***^	
Activation T_0_				0.430^***^
Stockholm	0.113	0.213	−0.083	0.012
Göteborg	0.006	−0.104	−0.178^*^	0.038
Sex	−0.202^*^	−0.255^*^	−0.089	−0.063
Age	0.010^**^	0.031^**^	0.003	0.004
Car			−0.359^***^	−0.184^*^
Public transport			−0.515^***^	−0.729^***^
Winter	−0.127	0.036	−0.002	−0.005
Sunshine	0.200^*^		0.094	−0.045
Temperature			0.094^**^	0.037
Wind speed			0.050	−0.034
Rain			−0.161^*^	−0.133
Wind speed × Public transport				0.461^***^
Sunshine × Slow modes			−0.448^***^	
Variance component (Adj *R*^2^)	0.373^**^	0.660^**^	0.105^***^	0.173^**^

* *p < 0.10*,

** *p < 0.05*,

*** *p < 0.01*.

Mood following the commute trip (valence and activation at *T*_*1*_) is strongly determined by mood at *T*_*0*_. Thus, people in a positive mood in the morning are still in a positive mood directly after the commute. As can be seen in Table 2, reported mood directly following the commute is associated with travel mode as well as some weather variables. Individual characteristics do not significantly determine mood reported directly after the commute, although participants living in Göteborg report a marginally significant lower valence after their commute. Participants commuting by car and public transport are in a less positive and activated mood directly after the commute than people commuting by slow modes. Commuting by public transport is associated with a less positive and activated mood than commuting by car. The effect is particularly pronounced for activation which implies that public transports users feel somewhat less alert and more bored following the commute.

Season (winter vs. summer) is not associated with reported mood directly following the commute. However, specific weather attributes have significant effects on mood. As reported in Table 2, a temperature higher than the monthly average is associated with an increase in valence directly after the commute. Rain or snow during the commute is associated with a decrease in valence directly after the commute. Thus, an increase in temperature makes commuters somewhat more glad and pleased whereas rain or snow makes them somewhat more sad and displeased. Table 2 also reports some differences due to travel mode. Contrary to expected, in sunshine commuting by slow modes (cycle or walking) is associated with less positive valence directly after the commute. Commuting by public transport is associated with higher activation directly after the commute during high wind speed.

### Travel satisfaction

We likewise used fixed-effects regression analyses to investigate the effects of season and weather attributes on travel satisfaction. The regression models were estimated separately for the three travel satisfaction dimensions (PDNA, PAND, and CE) directly after the commute (at *T*_*1*_). In the final models, all main effects of weather variables, but only interaction effects significant at *p* < 0.10 are reported in Table 3.

**Table 3:** **Unstandardized coefficients from the fixed-effects regression analyses of travel satisfaction (PDNA, PAND, CE) directly after the commute (*T*_*1*_)**.

	**Travel satisfaction (STS)**		
	**PDNA M *(SD)***	**PAND M *(SD)***	**CE M *(SD)***
Predictor	1.21 (1.23)	0.66 (1.20)	1.45 (1.04)
Intercept	0.538^**^	0.334	1.592^***^
Stockholm	−0.092	−0.064	−0.037
Göteborg	−0.237	−0.225	−0.069
Sex	−0.186	−0.093	−0.182^*^
Age	0.021^***^	0.020^***^	0.006
Car	−0.328^*^	−0.544^***^	−0.449^***^
Public transport	−0.450^***^	−0.763^***^	−0.622^***^
Winter	0.083	−0.040	−0.103
Sunshine	0.360^***^	0.171^*^	0.265^**^
Temperature	0.372^***^	0.042	0.021
Rain/snow	0.262^*^	−0.011	0.389^***^
Wind speed	0.076	0.219^**^	0.077
Temperature × Public transport	−0.251^*^		
Temperature × Slow modes	−0.345^***^		
Sunshine × Slow modes	−0.396^*^		−0.409^**^
Summer × Public transport × Rain/snow	0.550^*^		
Variance component (Adj *R*^2^)	0.482^***^	0.581^***^	0.335^***^

The CE scale assesses the cognitive (quality) evaluation of the commute, the PAND scale enthusiasm vs. boredom and the PDNA scale relaxation vs. stress.

* *p < 0.10*,

** *p < 0.05*,

*** *p < 0.01*.

The results displayed in Table 3 show that the measures of travel satisfaction measured by three different STS scales (CE, PAND, and PDNA) are significantly associated with different individual characteristics, commute attributes, and weather variables. Older people experience higher positive activation (enthusiasm) and positive de-activation (relaxation) than younger people. Men evaluate their commute as of less quality than women do. Public transport is associated with less quality, less enthusiasm, and less relaxation than car, which is associated with less quality, less enthusiasm, and less relaxation than slow modes. Travel satisfaction does not differ between seasons but is influenced by different weather variables. Sunshine and higher temperatures during the commute increase relaxation (PDNA), quality (CE), and marginally degree of enthusiasm (PAND). Also, wind speed increases enthusiasm (PAND). Rain increases quality (CE). Unexpectedly, commuting in sunshine by slow modes does not increase travel satisfaction as indicated by the two-way interaction. A three-way interaction indicates that people feel more relaxed (PDNA) when they commute by public transport in rain during summer.

## Discussion

This study aimed at investigating the extent to which season and weather influence travel satisfaction and mood experienced directly after a commute to work. We first note that with the exception of a nearly significant positive effect of sunshine on valence, the results showed no significant main effects of season and weather on mood before the commute. This is inconsistent with other research (e.g., Denissen et al., 2008). Perhaps we did not observe any effect because participants had not yet spent time outdoors. The effects of weather on mood are observed after the commute.

The effects of daily temperature and precipitation on mood after the commute are as expected. Higher temperatures made participants feel more pleased after the commute, whereas rain or snow had an opposite negative effect. Some travel mode interactions were also detected. Sunshine was unexpectedly found to have a significant negative effect on valence when cycling or walking. One possible explanation is that sunshine made those using slow modes too warm. Activation after the commute was only affected by travel mode, with slow modes leading to higher levels of activation. It was expected that active travel would have such an effect. A significant unexpected positive effect of wind speed on activation was found for public transport users. Being inside is perhaps beneficial when it is windy.

The effects of weather variables on travel satisfaction are similar to those obtained for mood following the commute. An additional finding is that sunshine positively influences both the affective and cognitive evaluation of the commute. Thus, sunshine makes people feel enthusiastic and relaxed directly after the commute and they evaluate the quality of the commute as more positive. This finding is consistent with research indicating that sunshine is associated with more positive mood (Kämpfer and Mutz, 2013). Slow modes eliminated the effect of sunshine on travel satisfaction, thus again suggesting that cycling and walking is less attractive during warmer temperatures. Contrary to St-Louis et al. (2014) we did not observe a season effect on travel satisfaction. This suggests that more research is needed into the season effects in different geographical and climatological settings.

Our findings have some implications for policy makers and transport planners. It is important to note that the effects of weather and season on travel satisfaction are limited, and mainly refer to feelings of relaxation during higher temperatures. This should however not be construed as an argument for policies aimed to mitigate negative weather or seasonal impact, such as providing shelter for public transport users or providing advance weather information. In Sweden, there are occasionally extreme weather conditions when shelter is absolutely necessary. Providing opportunities for people to experience a good weather by providing bicycle and walking lanes should be prioritized. Measures to minimize the effect of warmer temperatures can be electric bicycles, showers and changing facilities at workplaces and schools and walkways with roof between, for example, public buildings, and bus stops.

While our study delivers first insights into the season and weather impacts on travel-related mood and travel satisfaction, more research remains to be conducted. First, the set of weather variables can be extended to also include, for instance, humidity and air pressure. In addition, measurement may be extended to more periods during the year (fall, mid-summer, mid-winter), since these periods differ in terms of daylight during travel and average temperature from the time periods that we investigated. Another important extension would be to carry out similar studies in more diverse climatological conditions. Studies of weather and season effects on travel satisfaction and travel-related mood have taken place in cold or moderate climate zones (Canada, Sweden, Netherlands). It is likely that effects in warmer climates may vary considerably, and display different mode-specific effects, such as walking and cycling becoming much less attractive. Likewise, travel by air-conditioned vehicles would likely mitigate climate effects on travel satisfaction and mood in southern countries, as will sun protection for pedestrians and cyclists provided by buildings and vegetation.

## Ethics statement

The research were conducted in accordance with approved research protocols. The procedure ensured that participants were informed that confidentiality were maintained.

## Author contributions

All authors of the paper were involved in conception and design of the study, analyzing the data, and writing up the results into a paper. All authors agree to be accountable of all aspects of the work.

## Funding

Financial support was obtained through grant 2014-05335 from the Swedish Governmental Agency for Innovation Systems.

### Conflict of interest statement

The authors declare that the research was conducted in the absence of any commercial or financial relationships that could be construed as a potential conflict of interest.

## Footnotes

^1^In Friman et al. (Submitted) we report the results of an earlier wave using the same procedure and smartphone application. Here the procedure description is limited to the collection of the data used in the analyses, thus leaving out some information that can be found in the previous paper.

^2^A third mood questionnaire was administered one hour after the end of the commute. Results from this measure are not presented in this paper.

^3^Asking questions in a smartphone makes it necessary to decrease response burden. Therefore, we shortened the STS such that it consisted of only three scales with end-points defined by the adjectives that in the full-length STS are used to define the three scales for each dimension. Since the three scales used to measure each dimension in the STS have been shown to be strongly correlated (Friman et al., 2013), the shortened STS should not give different results. In indirect empirical support, Västfjäll and Gärling (2007) found that shortening the SCAS in the same way gave almost identical results to the full-length scale.

^4^Fixed effects regression analysis accounts for the fact that individuals may make multiple trips. Since we analyze mood at the level of trips, variance in the dependent variable may be associated with (in this case) the individual (e.g., due to characteristics such as age and gender) or the trip (e.g., due to trip characteristics such as travel mode, weather, and duration). Fixed effects regression analysis accounts for the different levels at which explanatory variables are specified. Not accounting for this may lead to biased results and overestimation of effects at the (in this case) individual level.
