# Supplementary material for: Season and Weather Effects on Travel-Related Mood and Travel Satisfaction
Source: Front Psychol. 2017 Feb 6;8:140. doi: 10.3389/fpsyg.2017.00140 (PMC5292578; doi:10.3389/fpsyg.2017.00140)
Supplement: Supplementary file 1 [file DataSheet1.docx]

**Appendix**

Table A1. Descriptive of wind in m/s across season during the data collections.

| **Wind m/s** | February/March | June | *Total* |
| --- | --- | --- | --- |
| 0 | 3.9% | 2.5% | *3.2%* |
| 1 | 7.4% | 7.1% | *7.3%* |
| 2 | 18.4% | 21.4% | *19.9%* |
| 3 | 12.8% | 22.1% | *17.4%* |
| 4 | 13.8% | 18.9% | *16.4%* |
| 5 | 23.4% | 17.5% | *20.5%* |
| 6 | 3.2% | 7.9% | *5.5%* |
| 7 | 10.6% | 0.7% | *5.7%* |
| 8 | 5.7% | 1.8% | *3.7%* |
| 9 | 0.7% | - | *0.4%* |
| *Total N* | *282* | *280* | *562* |

*Note: The grey area represents the cutoff point for a dummy variable in the forthcoming regression.*

Table A2. Descriptive of clouds across season during the data collections.

| **Clouds** | February/March | June | *Total* |
| --- | --- | --- | --- |
| Clear sky | 34.4% | 63.2% | *48.8%* |
| Occasional | 7.8% | - | *3.9%* |
| Scattered | 17.4% | - | *8.7%* |
| Broken clouds | 18.4% | 27.5% | *23.0%* |
| Cloudy | 22.0% | 9.3% | *15.7%* |
| *Total N* | *282* | *280* | *562* |

*Note: The grey area represents the cutoff point for a dummy variable in the forthcoming regression.*

Table A3. Descriptive of rainfall (in mm/half hour) across season during the data collections.

| **Rainfall (mm)** | February/March | June | *Total* |
| --- | --- | --- | --- |
| 0 | 68.1% | 74.3% | *71.2%* |
| 0.1 | - | 2.1% | *1.1%* |
| 0.3 | 1.4% | - | *0.7%* |
| 0.4 | 3.5% | - | *1.8%* |
| 0.5 | - | 2.9% | *1.4%* |
| 0.6 | 2.5% | - | *1.2%* |
| 1.1 | - | 1.1% | *0.5%* |
| 1.5 | - | 3.6% | *1.8%* |
| 2.6 | 2.5% | - | *1.2%* |
| 2.8 | 3.5% | 3.9% | *3.7%* |
| 3.3 | 1.8% | - | *0.9%* |
| 3.4 | 3.9% | - | *2.0%* |
| 3.8 | 4.6% | - | *2.3%* |
| 4.4 | 6.0% | - | *3.0%* |
| 5.7 | - | - | *1.4%* |
| 6 | 2.1% | - | *1.1%* |
| 9.2 | - | 2.1% | *1.1%* |
| 13.1 | - | 1.8% | *0.9%* |
| 25.3 | - | 5.4% | *2.7%* |
| *Total N* | *282* | *280* | *562* |

*Note: The grey area represents the cutoff point for a dummy variable in the forthcoming regression.*
